# Constitutively Elevated Blood Serotonin Is Associated with Bone Loss and Type 2 Diabetes in Rats

**DOI:** 10.1371/journal.pone.0150102

**Published:** 2016-02-23

**Authors:** Igor Erjavec, Tatjana Bordukalo-Niksic, Jelena Brkljacic, Danka Grcevic, Gordana Mokrovic, Maja Kesic, Dunja Rogic, William Zavadoski, Vishwas M. Paralkar, Lovorka Grgurevic, Vladimir Trkulja, Lipa Cicin-Sain, Slobodan Vukicevic

**Affiliations:** 1 Laboratory of Mineralized Tissues, Center for Translational and Clinical Research, School of Medicine, University of Zagreb, Zagreb, Croatia; 2 Department of Physiology and Immunology, School of Medicine, University of Zagreb, Zagreb, Croatia; 3 Laboratory for Neurochemistry and Molecular Neurobiology, Molecular Biology Department, Rudjer Boskovic Institute, Zagreb, Croatia; 4 Clinical Hospital Center Zagreb, Zagreb, Croatia; 5 Karos Pharmaceuticals, New Haven, CT, United States of America; 6 Department of Pharmacology, School of Medicine, University of Zagreb, Zagreb, Croatia; Max-Delbrück Center for Molecular Medicine (MDC), GERMANY

## Abstract

Reduced peripheral serotonin (5HT) in mice lacking tryptophan hydroxylase (TPH1), the rate limiting enzyme for 5HT synthesis, was reported to be anabolic to the skeleton. However, in other studies TPH1 deletion either had no bone effect or an age dependent inhibition of osteoclastic bone resorption. The role of 5HT in bone therefore remains poorly understood. To address this issue, we used selective breeding to create rat sublines with constitutively high (high-5HT) and low (low-5HT) platelet 5HT level (PSL) and platelet 5HT uptake (PSU). High-5HT rats had decreased bone volume due to increased bone turnover characterized by increased bone formation and mineral apposition rate, increased osteoclast number and serum C-telopeptide level. Daily oral administration of the TPH1 inhibitor (LX1032) for 6 weeks reduced PSL and increased the trabecular bone volume and trabecular number of the spine and femur in high-5HT rats. High-5HT animals also developed a type 2 diabetes (T2D) phenotype with increased: plasma insulin, glucose, hemoglobin A1c, body weight, visceral fat, β-cell pancreatic islets size, serum cholesterol, and decreased muscle strength. Serum calcium accretion mediated by parathyroid hormone slightly increased, whereas treatment with 1,25(OH)_2_D_3_ decreased PSL. Insulin reduction was paralleled by a drop in PSL in high-5HT rats. *In vitro*, insulin and 5HT synergistically up-regulated osteoblast differentiation isolated from high-5HT rats, whereas TPH1 inhibition decreased the number of bone marrow-derived osteoclasts. These results suggest that constitutively elevated PSL is associated with bone loss and T2D via a homeostatic interplay between the peripheral 5HT, bone and insulin.

## Introduction

Serotonin (5-hydroxytryptamine, 5HT) has multiple functions in peripheral organs acting via 15 transmembrane receptors. Centrally and peripherally synthesized 5HT functions independently due to the inability of 5HT to cross the blood-brain barrier. 5HT transport across the cytoplasmic membrane is regulated by the 5HT transporter (SERT or 5HTT), a member of the family of Na^+^/Cl^-^ dependent exchangers, which does not by itself induce any intracellular signaling. Recently, it was suggested that gut-derived 5HT suppresses osteoblast proliferation and that its synthesis is regulated via the low-density lipoprotein receptor-related protein 5 (LRP5) in the gut, but in osteoblasts 5HT acts in a non-LRP5 dependent manner to inhibit bone formation [1]. Also, transcription factors FOXO1, CREB and ATF4 have been identified as a molecular mode of intricate transcriptional machinery that confers the gut 5HT signal to inhibit bone formation without influencing osteoclast activity [2]. However, an association among LRP5 deficiency, circulating 5HT and bone loss has not been reproduced in mice with osteocyte-specific expression of inducible *Lrp5* mutations that cause high and low bone mass phenotypes in humans [3]. Further, association between circulating serotonin and bone mass has not been unequivocally confirmed in different mouse knockout models (global knockouts of TPH1 and TPH2, LRP5) [4–6]. De Vernejoul and colleagues revisited the bone phenotype in mice with genetic deletion of peripheral 5HT-synthesizing enzyme tryptophan hydroxylase-1 (*TPH1*^*-/-*^) and showed that osteoclasts synthesize 5HT which acts to induce osteoclast precursor differentiation in a local micro-serotoninergic system via a mechanism of RANKL-induced osteoclast formation [7].

There is controversy regarding the role of LRP5 in regulating peripheral serotonin levels and influence of peripheral serotonin on bone homeostasis [8–16]. Further, use of antidepressant medications, which act on serotonin system, has been shown to have impact on decreasing BMD and to increase risk for osteoporosis [17]. It should be mentioned, however, that methods of measurement of 5HT in the circulation vary in literature and differences in 5HT measurement should be taken into account when comparing different literature data.

Since the role of 5HT in bone metabolism remains poorly understood, we developed a novel rodent model of constitutionally altered 5HT homeostasis established by crossing Wistar rats selected for high or low platelet 5HT level (PSL) and the velocity of platelet 5HT uptake (PSU), representing the number of functional 5HT transporters. Bidirectional genetic selection of rats toward extreme activities of platelet 5HTT through successive generations, resulted in divergence into two discrete sublines with lifelong hyper- or hyposerotonemia as well as constitutional hyper- or hypoactivity of the serotonergic system in general [18]. We hypothesized that these constitutive differences in serotonergic phenotype might affect skeletal tissue. In these studies we demonstrated that rats with high PSL had an increased bone turnover with subsequent bone loss and developed a type 2 diabetes (T2D) phenotype.

## Materials and Methods

### Animals

Studies were performed on two sublines of the Wistar Zagreb 5HT (WZ-5HT) rats, with constitutively upregulated or downregulated platelet 5HT transporter and, consequentially, high or low platelet 5HT concentration (high-5HT and low-5HT subline), which was developed at the Rudjer Boskovic Institute as described previously [19,20]. In brief, males and females with the highest and the lowest values of platelet 5HT parameters, respectively, were chosen from a large base population of Wistar rats and mated together to generate high- and low-5HT sublines. Determination of platelet serotonin level (PSL) and platelet serotonin uptake (PSU) was performed in offspring of each breeding generation at approximately 4 weeks of age as previously described [18]. Divergence of mean values of platelet 5HT parameters stabilized after 5 generations at about 70% (low-5HT) and 150% (high-5HT) of the mean value of the starting population. Selective breeding was restarted *ab ovo* several times during the past decade with essentially the same final range of differences between 5HT sublines, confirming the reproducibility of the selection process. In this study female animals from five consecutive generations (F10 to F14) of selective breeding were used. They were housed three per cage under controlled conditions of temperature (23±2°C), humidity (55±10%) and light cycle (12h light/12h dark) with food (Mucedola) and water *ad libitum*.

In all experiments animals from both 5HT sublines were analyzed simultaneously. Experiments were performed in accordance with the NIH Guide for the Care and Use of Laboratory Animals and research protocols were approved by Croatian Ministry of Agriculture and ethical committee of the School of Medicine, University of Zagreb (525-1010255-12-2).

### Muscle strength test

To test the muscle strength in young and aged rats from both 5HT-sublines string test was employed. The string test measures the length of time the rat hung from a horizontal wire. In three consecutive trials (60 sec, separated by 10 min intervals) the animals were suspended by their forepaws on a horizontally stretched wire (2 mm in diameter, 40 cm in length, 80 cm from the carpeted floor) and the latency to fall time was measured with a stopwatch. The averaged measurements were expressed per 100 g of body weight.

### Gut 5HT immunohistochemistry

Isolated gut tissue samples from duodenum were fixed in 10% paraformaldehyde and were embedded in paraffin blocks afterwards. Sections were cut at 5 μm thickness and placed on charged slides. Slides were deparaffinized in xylene and rehydrated in a descending series of ethanol, and the 1× PBS. Rehydrated slides were incubated in 3% H_2_O_2_ in methanol to quench the endogenous peroxidase activity. Slides were then incubated with rabbit polyclonal antibody against 5HT (Immunostar, 20080) diluted 1:1000 in 1× PBS overnight at +4°C in a humidifying chamber [21,22]. The reaction was detected using Histostain SP kit (Invitrogen) while staining was visualized using AEC chromogen. Slides were counterstained in hematoxylin and mounted using ClearMount (Invitrogen).

### Micro CT analyses

Computed tomography (CT) analyses were completed by using SkyScan 1076 (SkyScan) micro CT scanner. Bone samples were scanned at 50kV/200μA which corresponds to an 18μm spatial resolution throughout 198° with a 0.6° rotation step. Trabecular bone was assessed by CTAn software (Skyscan) where at the peripheral skeleton distal femur site was analyzed while the third lumbar vertebra was chosen for the axial skeleton analysis [23]. To analyze the fat tissue in rats a visceral region corresponding to 3–5 lumbar vertebrae was scanned *in vivo* at 70kV/140μA which corresponds to a 35μm spatial resolution throughout 360° with a 0.7° rotation step. Data analysis was carried out throughout the whole scanned area of the animal using CTAn (SkyScan) software.

### Histomorphometry

For dynamic histomorphometric analysis, calcein at a concentration of 10 mg/kg (Sigma-Aldrich) dissolved in 2% Na_2_HPO_4_ was injected intraperitoneally seven and two days prior to experiment termination. Extracted bones were fixed in 10% buffered formaldehyde and processed as previously described [24,25]. Histomorphometric analysis was executed using Osteomeasure XP (Osteometrics) software.

### Biochemical analyses

Methods for blood sampling, preparation of platelet-rich-plasma (PRP), and determination of PSL (spectrophotofluorimetrically) and PSU (radiochemically) in the same sample, were described previously [18]. For repetitive sampling, rat blood samples were collected from retro-orbital sinus into EDTA-coated vacutainers (BD). Blood was centrifuged within 30 min of sample collection, 15 min at 1000×g at 4°C. Plasma was separated, aliquoted immediately and stored at -80°C. For serum sampling, rat blood samples were collected in tubes without anticoagulant and centrifuged after 30 min for 15 min at 1000×g at 4°C. Plasma or serum levels biochemical markers were measured by commercially available ELISA kits: C-telopeptide, 1,25(OH)_2_D_3_, insulin (MyBiosource), osteocalcin (Takara), fibroblast growth factor 23 (FGF23, Kainos Pharmaceuticals), PTH (Immutopics). In all assays, plasma samples from both rat sublines were analyzed in parallel. The concentration of 5HT in the primary cell culture supernatant was determined by the Serotonin High Sensitive ELISA kit (IBL).

Blood biochemical parameters from high- and low-5HT animals were measured using the clinical chemical analysis machine Roche Cobas 6000 (Roche). All the original reagents, standards and controls were obtained from Roche. Calcium levels were measured using o-cresophtalein and serum phosphate concentration by the molbidenium method. Total cholesterol in the plasma was measured using the cholesterol oxidase method, triglycerides were measured using the enzymatic reaction, while HDL was measured directly. Haemoglobin A1c was measured by the turbidimetric method.

### Measurement of intestinal 5HT and 5HIAA content

Weighed samples of gut mucosa (20–50 mg) were added to 2 mL flat bottom microfuge tubes. The tissue samples were homogenized in 250 μL of buffer containing 0.1M sodium actetate, 20 mM sodium bisulfate, 0.3M trichloroacetic acid, 10 mM EDTA, and 50 mM ascorbic acid. Following homogenization, the tubes were centrifuged at 18,000×g for 30 minutes at 4°C. The supernatant was carefully removed and transferred to a glass vial for measurement of 5-HT and 5-HIAA via HPLC-MS. Sample extracts were further diluted with acidified methanol containing internal standard and chromatographed using reverse phase liquid chromatography on a Perkin Elmer 200 series HLPC using a Phenomenex Polar-RP 80A column (100×3.0 mm; Phenomenex). Detection was by tandem mass spectrometry using a Sciex API-3000 tandem mass spectrometer. LC/MS/MS data was acquired using Analyst v1.6 software. 5HT and 5HIAA levels were reported in ng/mL of extraction volume and converted to pmol/mg tissue weight.

### Glucose and insulin tolerance test

A glucose tolerance test (GTT) and an insulin tolerance test (ITT) were performed in 6 month old females from both rat sublines (n = 10). In the GTT, animals were treated with i.p. injection of glucose (1 g/kg) after an overnight fast, and blood glucose levels were monitored every 30 minutes for 2 hours. For the ITT, after a 5 h fast, animals were injected intraperitoneally with 1 U/kg insulin (Eli Lilly) and glucose was monitored every 30 minutes.

### Pancreatic β-cell insulin immunohistochemistry

5 μm-thick deparaffinized sections were subjected to heat induced epitope retrieval procedure (HIER) in a citrate buffer (Dako) using a microwave. To eliminate endogenous peroxidase activity, the sections were pretreated at room temperature with 3% H_2_O_2_ in methanol for 10 minutes. Sections were then incubated with the primary antibody against insulin (Santa Cruz, sc-9168) diluted 1:100 in 1× PBS overnight at +4°C in a moist chamber [26]. The reaction was detected using Histostain SP kit (Invitrogen) while staining was visualized using AEC chromogen. Slides were counterstained in hematoxylin and mounted using ClearMount (Invitrogen). Each slide was analyzed by microscope (Olympus Provis, Campbell) and photographed (magnification 40×) covering its entire surface. For the determination of beta islet size, number and diameter pancreatic sections stained for insulin and hematoxylin were analyzed. An islet was defined as a cluster of four or more insulin-positive cells. After careful assessment of the entire pancreatic section, five representative islets from five different microscopic fields from five paraffin blocks randomly selected from each group (5 rats from each group) were identified and islet size and diameter were measured. The computed morphometric image analysis and measurements of the digitalized images were carried out using Sform software (Vams).

### Pharmacological treatment of animals

Animals from both rat sublines were divided into three groups (n = 7) and treated for 7 days with fluvoxamine (20 mg/kg/day i.p.; Duphar-Solvay), parathyroid hormone (PTH; 10 μg/kg/day s.c.; Tocris) and 1,25(OH)_2_D_3_ (1 μg/kg/day s.c.; Tocris). Blood samples were taken prior to treatment and 24 h after the last injection for determination of PSL and insulin. In PTH and 1,25(OH)_2_D_3_ treatments, additional blood samples, for serum Ca^2+^ measurements, were taken 2 h after last injection.

For induction of diabetes, rats from the high-5HT subline (n = 11) were treated with a single i.p. injection of streptozotocin (STZ; 80 mg/kg; Sigma-Aldrich). Untreated high-5HT rats (n = 8) served as controls. Blood samples for determination of PSL and plasma insulin level were taken prior to the treatment and 6 weeks after STZ injection. During the 6 week-period, blood glucose levels were determined every 7 days.

For pharmacological inhibition of 5HT synthesis, 12-months old animals from the high-5HT subline (n = 6) were treated with 25 mg/kg of LX1032, a specific inhibitor of TPH1 enzyme [27], which was given during 36 days by oral gavage daily. LX1032 was prepared according to the procedure previously described (WO 2009/029499 patent). Untreated high-5HT rats of the same age (n = 7) served as controls. At the end of the treatment, PSL was determined, animals were sacrified and bone parameters were measured by microCT.

### Preparation of primary rat osteoblast culture from mesenchymal stem cells

Bone marrow from femurs and tibiae was collected by flushing with α-modified essential medium (α-MEM, Lonza) through sterile needle. After centrifugation of 5 min at 300×g, cell suspension was filtered through cell strainer. After counting, cells were plated at a density of 1×10^6^ cells/mL/well in 24-well culture plates (Falcon), in α-MEM supplemented with 1% penicillin and streptomicin and 10% dialyzed fetal bovine serum (FBS; Invitrogen), which does not contain 5HT. On day 4, half of the medium was changed. On day 7, medium was changed completely and supplemented with 10 mM of β-glycerophosphate and 50 μg/mL of ascorbic acid (Sigma-Aldrich). Half of the medium was replaced every third day. At the end of the culture (19 days), cells were rinsed in PBS and lysed in TRIreagent for RNA isolation. Differentiation of cells into osteoblasts was confirmed by staining cells on alkaline phosphatase (Sigma-Aldrich).

In some experiments, osteoblasts were treated with 5HT (Sigma-Aldrich) in final concentration of 50 nM, insulin (Eli Lilly) in final concentration of 1 μM, or their combination (n = 3 wells/treatment group). Substances were added to the medium from day 7 till end of the culture.

### Preparation of primary rat osteoclast culture

Suspension of bone marrow cells was prepared as described above. After counting, cells were plated at a density of 7.5×10^5^ cells/0.5 mL/well in 48-well culture plates (Falcon), in α-MEM with 1% penicillin and streptomicin and 10% dialyzed FBS, supplemented with 20 ng/mL of RANKL and M-CSF (R&D). Medium was changed at day 3, and at day 4, cells were rinsed in PBS and lysed in TRIreagent (Ambion) for RNA isolation. Differentiation into osteoclasts was confirmed by TRAP staining (Sigma-Aldrich) and counting under microscope.

In some experiments, osteoclasts were treated with 5HT (Sigma-Aldrich) in final concentration of 50 nM, insulin (Eli Lilly) in final concentration of 1 μM, LX1032, in final concentration of 5 nM, or their combination (n = 3 wells/treatment group). Substances were added from the beginning of the culture.

### Gene expression analyses

Total RNA from cell cultures was isolated using TRIreagent according to manufacturer's instructions. For measurement of the gut *Tph1* and *5Htt* expression in rats, animals were euthanized and approximately 50 mg tissue from duodenum was homogenized in 1 mL TRIreagent. 1 μg of total RNA was transcribed into cDNA using high-capacity cDNA reverse transcription kit (Applied Biosystems) and oligo-dT primers for mRNA transcription. For gene expression analyses quantitative real-time PCR was performed on a Light Cycler (Roche) using Fast Start DNA SYBR Green Master Plus (Roche). The comparative CT method (ΔΔCT) was used for relative quantification of gene expression [28]. Sequences of primers used are presented in Table 1. For normalization of expression level *β-actin* was used as a housekeeping gene.

**Table 1:** Primer sequences used in RT-PCR analysis.

Gene	Forward	Reverse
5HTT	5'-TCTGAAAAGCCCCACTGGACT-3'	5'-TAGGACCGTGTCTTCATCAGGC-3'
TPH1	5'-AGCATAACCAGCGCCATGAA-3'	5'-GGCATCATTGACGACATCGAG-3'
5HT-1B	5'-TGGCGTCAAGCCAAAGCGGA-3'	5'-AACTGGGCTCGGGTCAAGCG-3'
5HT-2A	5'-TTCACCACAGCCGCTTCAA-3'	5'-ATCCTGTAGTCCAAAGACTGGGATT-3'
5HT-2B	5'-GGCTGATTTGCTGGTTGGATTG-3'	5'-GGGCCATGTAGCCTCAAACATG-3'
MAO-A	5'-GGAGAAGCCCAATCTCGCAGGC-3'	5'-GGGAATGCACCACGGAATGGGT-3'
osteocalcin	5'-AAGCCCAGCGACTCTGAGTCT-3'	5'-CCGGAGTCTATTCACCACCTTACT-3'
alkaline phosphatase	5'- TGAATCGGAACAACCTGACTGA-3'	5'-TTCCACTAGCAAGAAGAAGCCTTT-3'
TRAP	5'-ACGCCAATGACAAGAGGTTC-3'	5'-AGGTGATCATGGTTTCCAGC-3'
cathepsin K	5'-AGACGCTTACCCGTATGTGG-3'	5'-CACTGCTCTCTTCAGGGCTT-3'
β-actin	5'-GCGCAAGTACTCTGTGTGGA-3'	5'-ACATCTGCTGGAAGGTGGAC-3'

### Data analysis

Gene expression data were analyzed by fitting general linear mixed models (GLMMs) to account for potential correlations between simultaneously analyzed genes. Data from the *in vivo* pharmacological intervention experiments, GTT and ITT were also analyzed using GLMMs to account for autocorrelations (repeated measurements). Relationships between different biochemical markers (serum calcium, PSL, insulin) were explored by fitting generalized additive models (GAMs) for non-parametric regression with cubic smoothing splines and were analyzed by fitting GLMMs. All other data were analyzed using univariate (independent t-test) or multivariate (general linear models, GLMs) tests, as appropriate. Differences between rats of a particular 5HT subline but of different age were considered to illustrate age-dependent changes within the subline. When needed, data were ln-transformed. Relative mean differences are expressed as “fold difference” or “percent difference” and are derived from geometric means ratios or from least-square means of the non-transformed data as 100*[(mean A − mean B)/mean B]. Adjustment for multiplicity was performed by the simulation method or by setting the critical alpha level at 0.025 when simulation method was considered too conservative. Analyses were performed by SAS for Windows 9.3 (SAS Inc.).

## Results

### 5HT animal model

**Fig 1:**
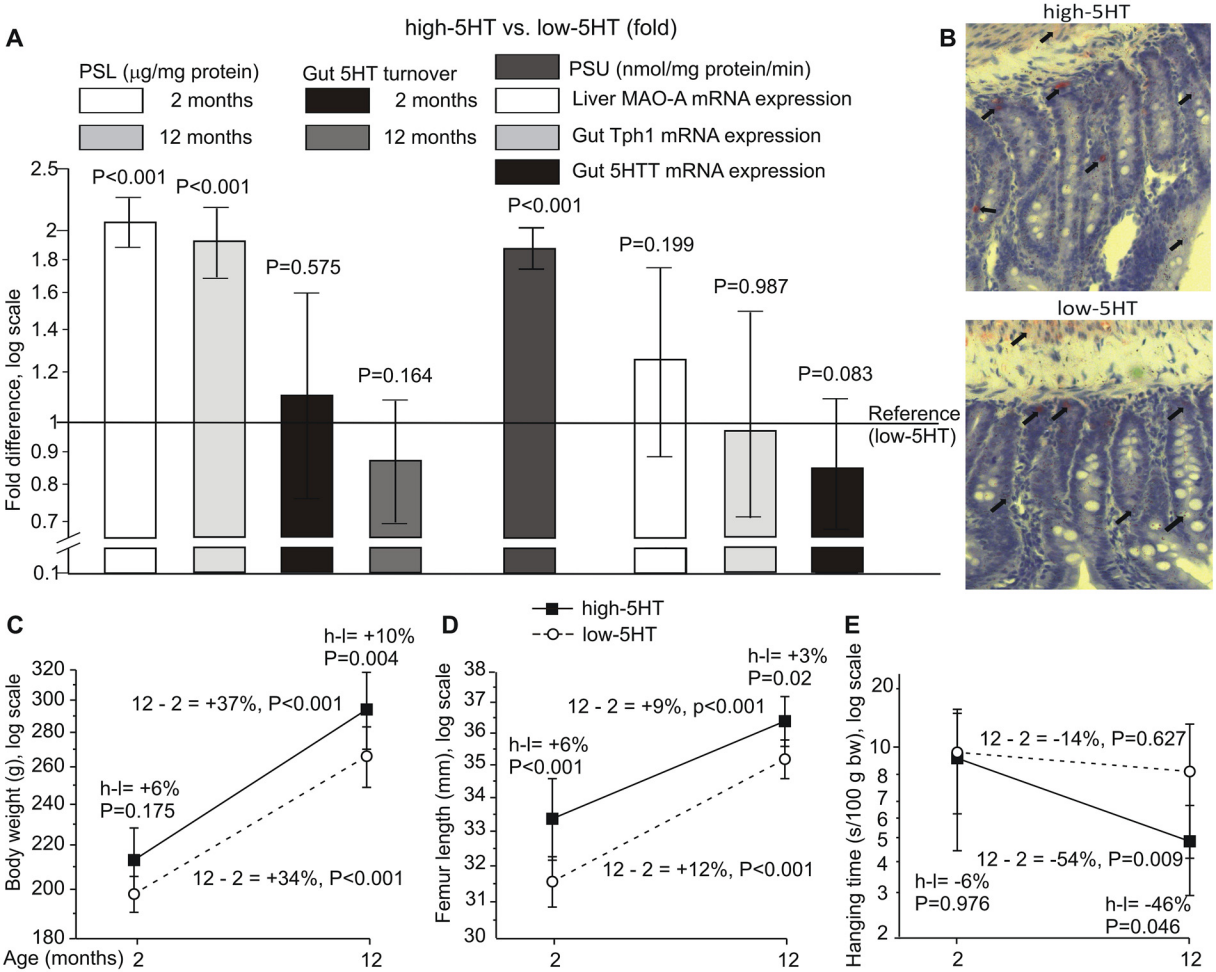
Physiological characteristics of rats from high-serotonin (5HT) and low-5HT sublines. A. Indicators of 5HT homeostasis shown as “fold difference” between high- and low-5HT animals with 95% confidence intervals. Reference values were (mean±SD): a) for platelet serotonin level (PSL) 0.80±0.08 μg 5HT/mg platelet protein; b) for platelet serotonin uptake (PSU) 0.69±0.07 nmol 5HT/mg platelet protein/min. Rats were 2 months (PSU measurements) and 12 months (gut *Mao-A*, *Tph1* and *5HTT* expression) of age. PSL and gut 5HT turnover data are given for animals of 2 and 12 months of age. B. No difference in 5HT production and storage in the gut was observed between high- and low-5HT rat sublines. 5HT visualized by using immunohistochemistry was documented at 40× magnification and is depicted by black arrows. C-E. Physical characteristics of high-5HT and low-5HT animals (mean±SD). High-5HT animals are represented by black squares, low-5HT animals by open circles. C—body weight; D—femur length; E—hanging time in the string test (mean values from three 60-sec trials separated by 10-min intervals). Relative differences (%) are shown for subline*age interaction contrasts. P-values were adjusted for multiple comparisons (n = 6–15 rats/group). H-L indicates a difference between high-5HT and low-5HT animals. 12–2 indicates a difference between 12 months and 2 months old animals. Mao-A—monoamine oxidase A; Tph1 –tryptophan hydroxylase 1; 5HTT—serotonin transporter

In all experiments rats from high-5HT and low-5HT sublines differed by approximately 2-fold in their platelet serotonin level (PSL) notwithstanding sex or breeding generation. Differences in PSL were present in both young and aged animals. The gut 5HIAA/5HT ratio, an indicator of 5HT turnover and gut metabolism (Fig 1A), and immunohistochemical analysis of the gut 5HT content (Fig 1B) were not different between the rat sublines. High-5HT animals had an elevated velocity of the platelet 5HT uptake (PSU) as compared to low-5HT rats. However, the gut gene expression of *Tph1*, *Mao-A* and *5HTt* were similar in both sublines (Fig 1A). Body weight (Fig 1C) and femur length (Fig 1D) increased in both sublines between the age of 2 and 12 months with consistently higher values in high-5HT rats. The muscle strength, as assessed by the string test (Fig 1E), progressively decreased in high-5HT rats.

### 5HT and bone volume

**Fig 2:**
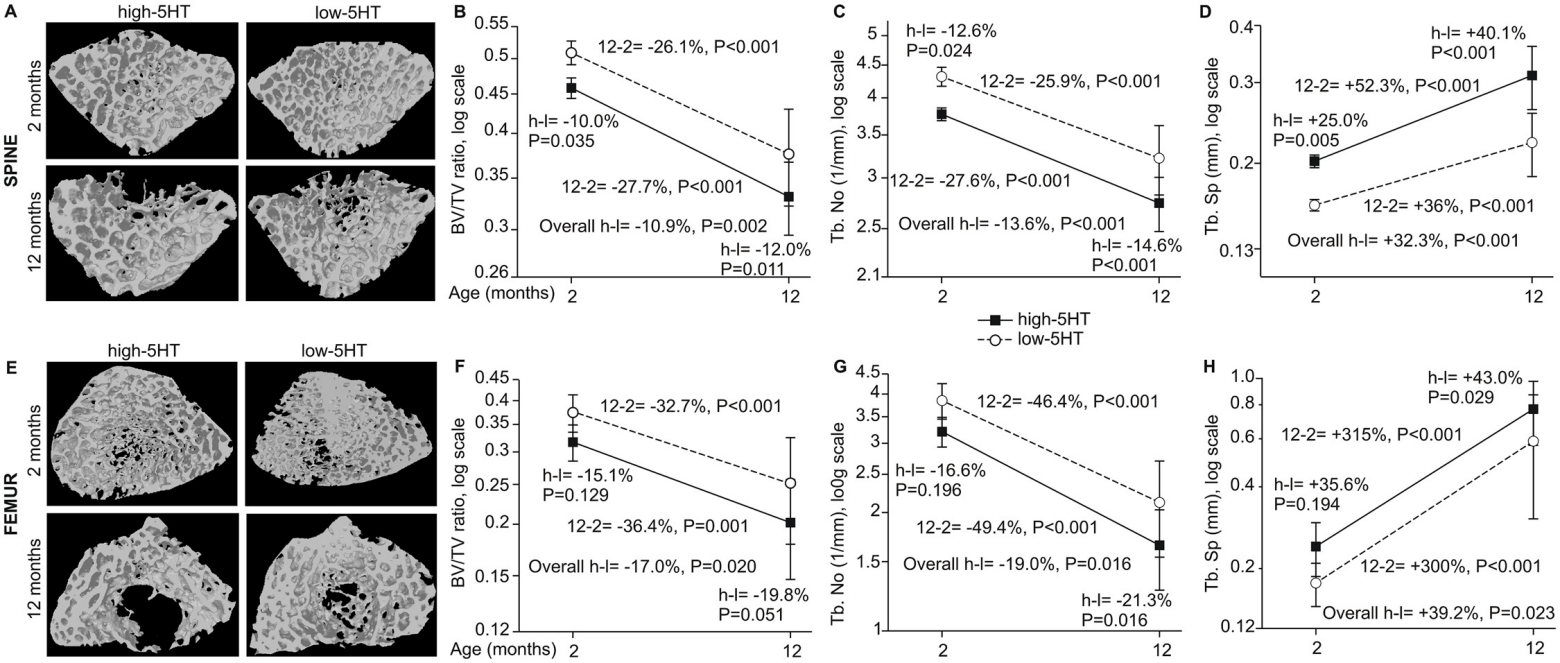
3D model of trabecular bone reconstructed from μCT images for lumbar spine and distal femur in high-5HT and low-5HT rats at 2 and 12 months of age. A. Spine—μCT images. B-D. Spine—morphometric indices (mean±SD). E. Femur—μCT images. F-H. Femur—morphometric indices (mean±SD). Shown are relative differences (%): H-L (high-5HT vs. low-5HT animals) at different age; 12–2 months for high and low 5HT animals (n = 8–14 rats/group). Depicted are relative differences (%): for the overall difference between the high-5HT and low-5HT sublines, P<0.05 is considered significant; for contrasts between sublines at a given age and between different ages within the same subline, P<0.025 is considered significant.

We analyzed the trabecular bone volume (BV/TV), separation (Tb.Sp) and number (Tb.No) in the lumbar spine (Fig 2A) and distal femur (Fig 2E) of animals from both 5HT sublines. The lumbar spine BV/TV ratio (Fig 2B) and Tb. No (Fig 2C) decreased between 2 and 12 months of age in both 5HT sublines, with values consistently lower in high-5HT rats. At the same time, Tb.Sp (Fig 2D) increased in both sublines. Identical age or subline-related patterns were observed at the site of distal femur (Fig 2E–2H). With ageing, bone volume differences between sublines were more pronounced.

### Increased remodeling rate in high-5HT rats

**Fig 3:**
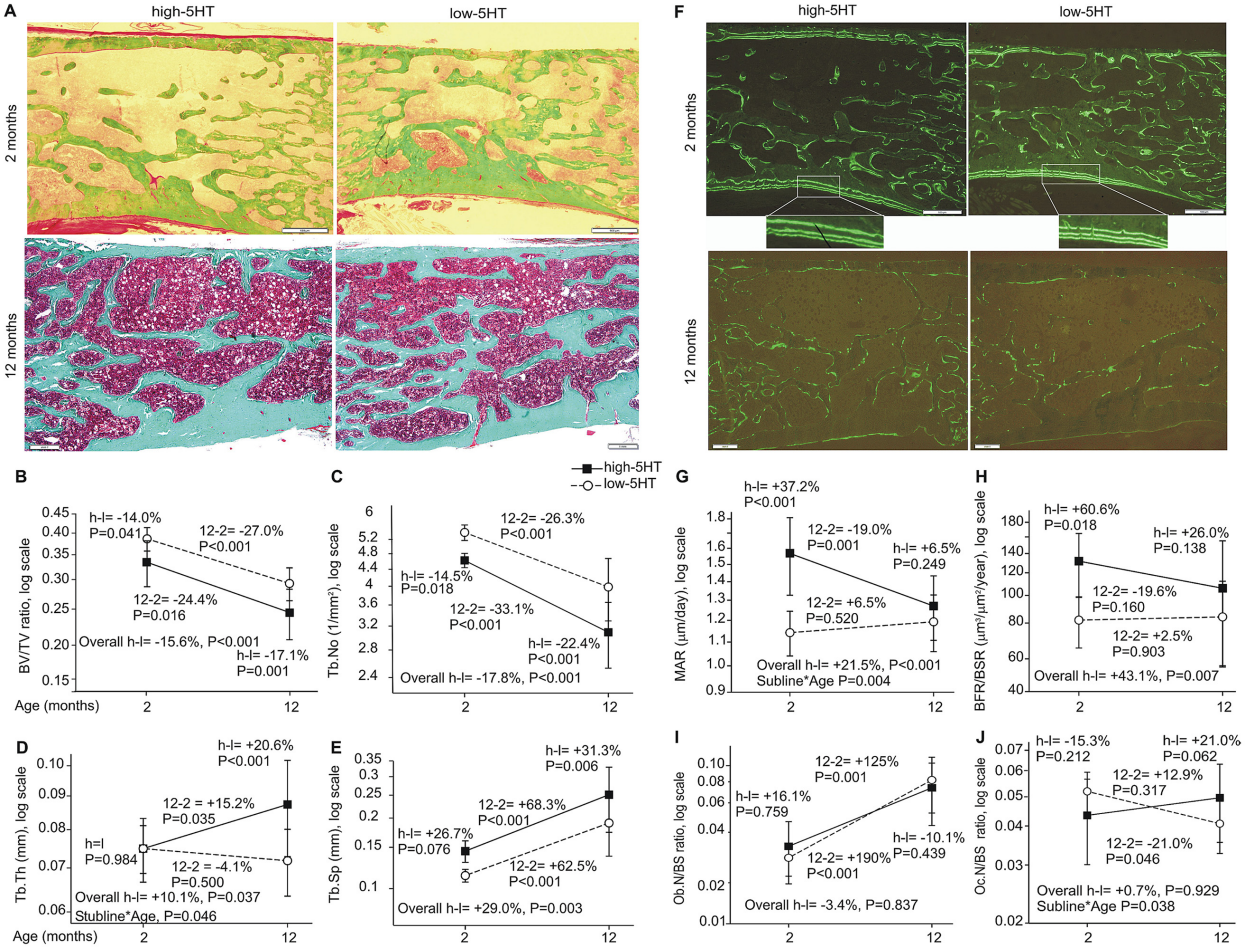
Histomorphometric analysis of the lumbar spine in high-5HT and low-5HT rats at 2 and 12 months of age. A-E Light microscopy and static morphometric parameters. F-G Fluorescent microscopy and dynamic morphometric parameters. I. Osteoblast numbers. J. Osteoclast numbers. Data are mean+/-SD (n = 8–14 rats/group). Depicted are relative differences (%): for the overall difference between the high-5HT and low-5HT sublines (h-l) and for the subline*age interaction (depicted only where significant), P<0.05 is considered significant; for contrasts between sublines at a given age and between different ages within the same subline, P<0.025 is considered significant. Boxes represent 500 μm.

Bone histomorphometry of the lumbar spine confirmed the results obtained by micro CT (Fig 3A and 3F). High-5HT subline had lower BV/TV, Tb.No and higher Tb.Sp in young and aged animals (Fig 3B, 3C and 3E). Dynamic histomorphometry showed an increased osteoblast activity via mineral apposition rate (MAR) (Fig 3G) and bone formation rate (BFR) (Fig 3H) in young high-5HT rats, but these differences were not found in aged animals. The osteoblast number (Ob.N/BS) was similar in young animals from both sublines (Fig 3I). Number of osteoclasts did not significantly differ between the sublines irrespective of their age (Fig 3J).

### Serum biochemical profile

**Fig 4:**
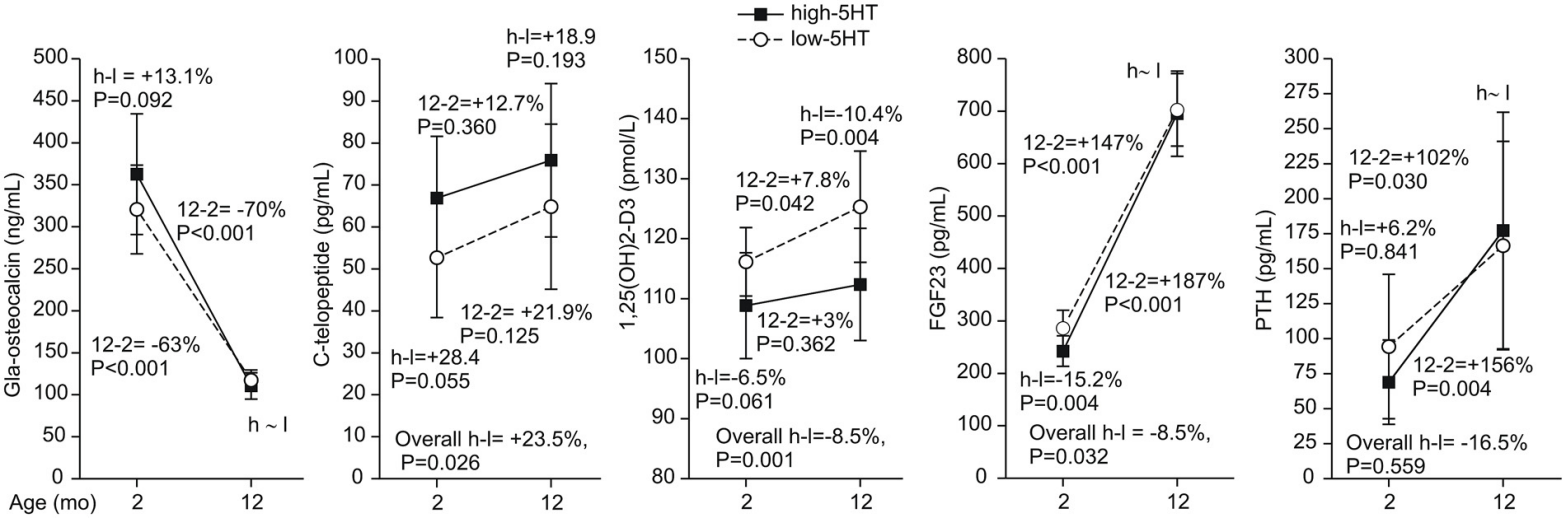
Bone biomarkers and hormones in high-5HT and low-5HT rats. Depicted are relative differences (%): for the overall difference between the high-5HT (H) and low-5HT (L) sublines, P<0.05 is considered significant; for contrasts between sublines at a given age and between different ages within the same subline, P<0.025 is considered significant (n = 5–10 rats/group). H-L indicates a difference between high-5HT and low-5HT animals. 12–2 indicates a difference between 12 months and 2 months old animals.

To further explore the relationship between 5HT and bone loss, we measured the bone remodeling markers and related calciotropic hormones. High-5HT rats had higher plasma C-telopeptide level and lower FGF23 at younger age (2 months old) and lower 1,25(OH)_2_D_3_ at older age (12 monhts old) whereas PTH and γ-decarboxylated osteocalcin concentrations did not differ between 5HT sublines irrespective of age (Fig 4). With ageing, osteocalcin decreased and C-telopeptide increased in both sublines confirming the morphometric analyses (see Figs 2 and 3).

### Development of a type 2 diabetes phenotype in high-5HT animals

**Fig 5:**
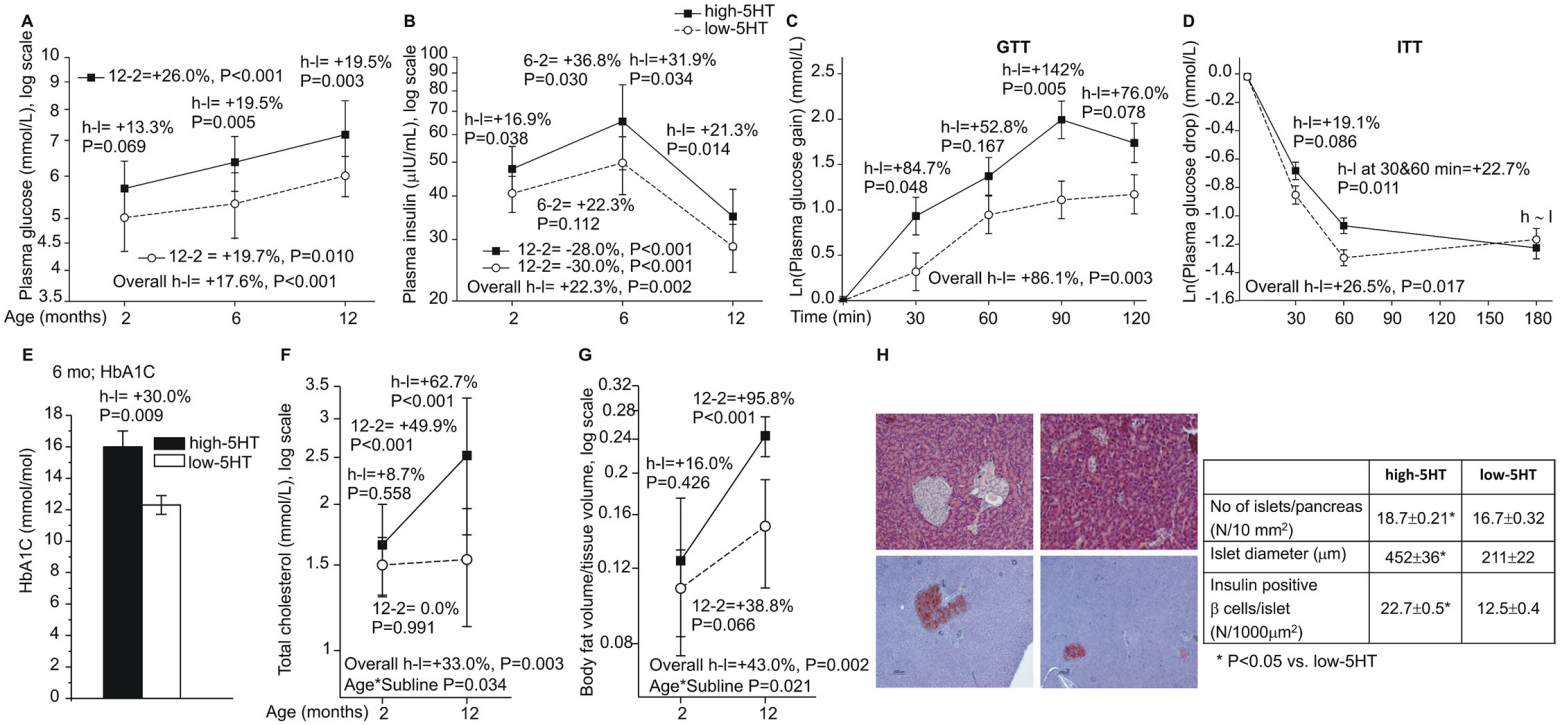
Development of glucose and lipid metabolism alterations with age in high-5HT and low-5HT rats. A-B. Plasma glucose and insulin levels (mean±SD, n = 7–24). C-D. Glucose metabolism functional tests in 6 months old animals. Data are least square mean±SE gain (GTT) or drop (ITT) in glucose levels (n = 8–10). E. Plasma HbA1c in 6 months old rats (mean±SD, n = 3). F-G. Indicators of lipid metabolism in 2 and 12 months old rats (mean±SD, n = 6–10). H. Pancreatic beta islet analysis in high-5HT and low-5HT rats (n = 6). Depicted are relative differences (%): for the overall difference between the high-5HT and low-5HT sublines (h-l), and for subline*age interaction (indicated only where significant), P<0.05 is considered significant; for contrasts between sublines at a given age (or time in GTT/ITT), and between different ages within the given subline, P<0.025 is considered significant. Analysis of GTT and ITT data was adjusted for baseline glucose levels. GTT- glucose tolerance test; ITT—insulin tolerance test.

Changes in plasma glucose (Fig 5A) and insulin levels (Fig 5B) over time resembled the occurrence of a type 2 diabetes (T2D) phenotype, and were consistently more extensive in high-5HT rats. At 2 months, and more pronounced at 6 months, high-5HT animals developed a glucose intolerance and insulin resistance, as evidenced by GTT (Fig 5C) and ITT (Fig 5D) experiments. In addition, the increased serum hemoglobin A1c (Fig 5E) indicated a shift in the glucose metabolism. In support, increased total serum cholesterol (Fig 5F) and visceral fat (Fig 5G) with age were considerably more pronounced in high-5HT rats, which also had an increased number and size of β-cell pancreatic islets (Fig 5H). These data suggest a causal relationship between the increased circulatory 5HT and the plasma insulin levels.

### Experimental interventions

**Fig 6:**
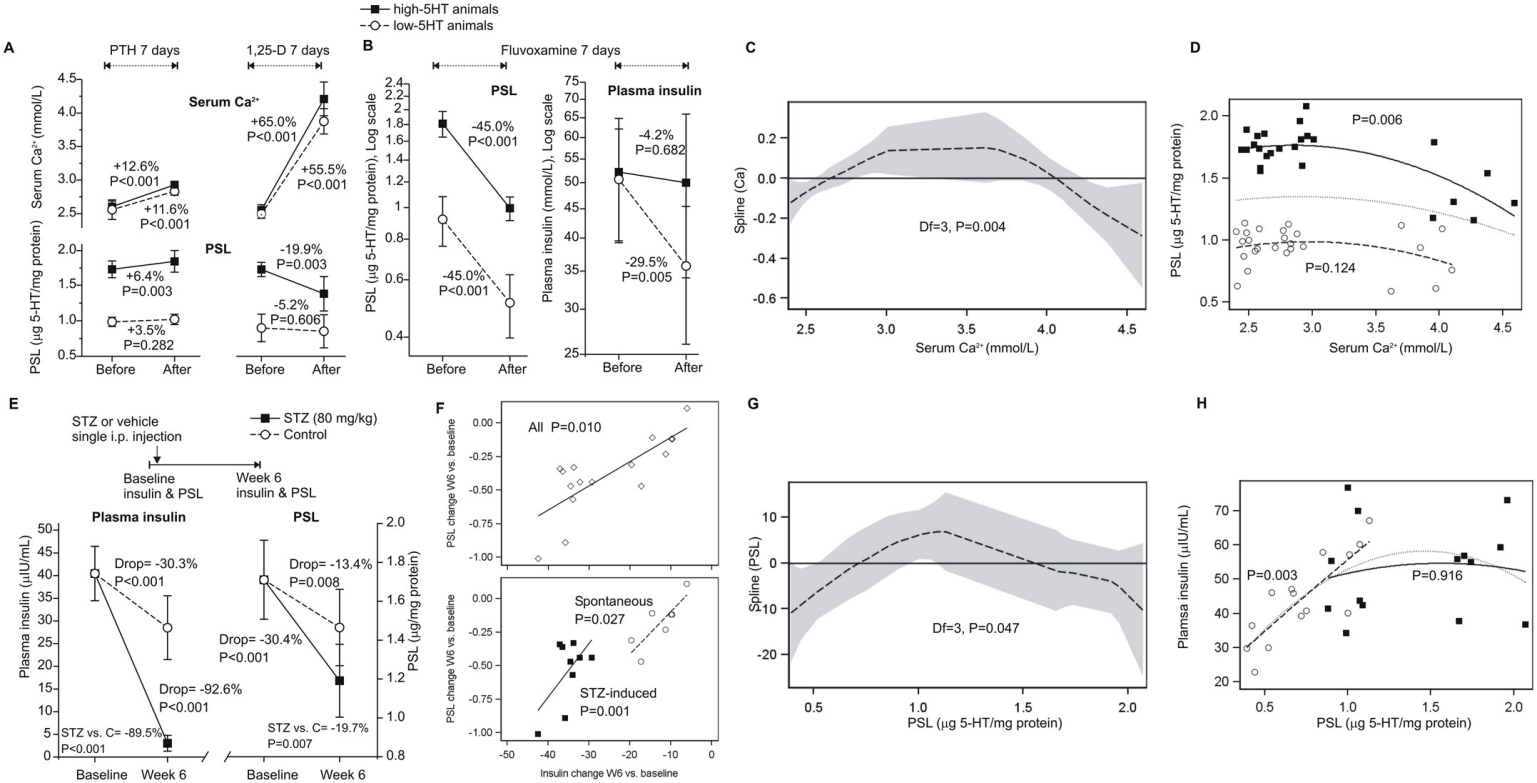
Effects of pharmacological interventions on serum Ca^2+^, PSL and plasma insulin levels in high-5HT and low-5HT rats, and relationships between them. A. Effects of a 7-day treatment with 10 μg/kg/day PTH or with 1 μg/kg/day 1,25(OH)_2_D_3_ on serum Ca^2+^ and PSL (mean±SD, n = 6–7 animals/group). B. Effects of a 7-day treatment with 20 mg/kg/day fluvoxamine on PSL and plasma insulin (mean±SD, n = 6–7 rats/group). C. Relationship between serum Ca^2+^ (independent) and PSL (dependent): exploratory non-parametric regression with a cubic spline smoother (dashed line; shaded area = 95% confidence interval) indicated a quadratic Ca^2+^- PSL relationship (deviance Chi^2^ = 13.9, DF = 3, P = 0.004). D. Relationship between serum Ca^2+^ and PSL: a mixed-effect model (repeated measures, two experiments) with quadratic Ca^2+^ (model fit quadratic Ca^2+^ -2ResLL = -25.3; AIC = -19.3 vs. -2ResLL = -31.9, AIC = -25.9 for linear Ca^2+^) and calcium*subline interaction term (P = 0.021). High-5HT rats = black squares (data) and a full line (fit) (coeff. = -0.028, SE = 0.011); low-5HT rats = open circles and a dashed line (coeff. = -0.006, SE = 0.005). Dotted line = quadratic fit for overall data (coeff. = -0.017, SE = 0.007; P = 0.008). E. Effect of streptozotocin (STZ)-induced diabetes on plasma insulin and PSL in high-5HT animals. F. Relationship between PSL and plasma insulin in a streptozotocin-induced diabetes model. G-H. Relationship between PSL (independent) and plasma insulin (dependent): exploratory non-parametric regression with a cubic spline smoother (dashed line; shaded area = 95% confidence interval) indicated a quadratic PSL—insulin relationship (deviance Chi^2^ = 7.9, DF = 3, P = 0.047). Exploratory non-parametric regression indicated a quadratic Ca^2+^- PSL and quadratic PSL—insulin relationship.

To further explore the relationship between calciotropic hormones, insulin and 5HT, rats were treated for 7 days with PTH, 1,25(OH)_2_D_3_ or fluvoxamine, a 5HT re-uptake inhibitor. PTH and 1,25(OH)_2_D_3_ in particular, increased serum calcium in both sublines (Fig 6A). In high-5HT rats, this was paralleled by a PSL increase in response to PTH (a modest increase in serum calcium) and a decrease in response to 1,25(OH)_2_D_3_ (a profound increase in serum calcium) (Fig 6A). Fluvoxamine lowered PSL in both sublines. This was paralleled by a drop in plasma insulin in low-5HT, but not in high-5HT animals (Fig 6B). Exploratory regression indicated a quadratic relationship between serum calcium levels (independent) and PSL (dependent) (Fig 6C), with a slight increase in PSL with increasing calcium at lower calcium levels and a decrease in PSL with increasing calcium at the levels above 3.5 mM (Fig 6D). To further explore the relationship between 5HT and insulin, high-5HT rats received a single injection of streptozotocin (STZ) resulting in a several fold reduction of the insulin level, paralleled by a significant decrease in PSL (Fig 6E). In control animals, insulin moderately declined, which was also accompanied by a discrete PSL decline (Fig 6F). A greater insulin decline was associated with a greater PSL decline (Fig 6H). This novel association between PSL and plasma insulin was additionally confirmed by correlation analysis between these two parameters. A quadratic relationship was indicated between PSL (independent) and plasma insulin (dependent) (Fig 6G). A mixed model revealed a significant association between PSL and insulin only in low-5HT rats (PSL×subline interaction): insulin increased with increasing PSL in the range of PSL values seen in the low-5HT subline and plateaued at high PSL levels as seen in the high-5HT subline (Fig 6H).

**Fig 7:**
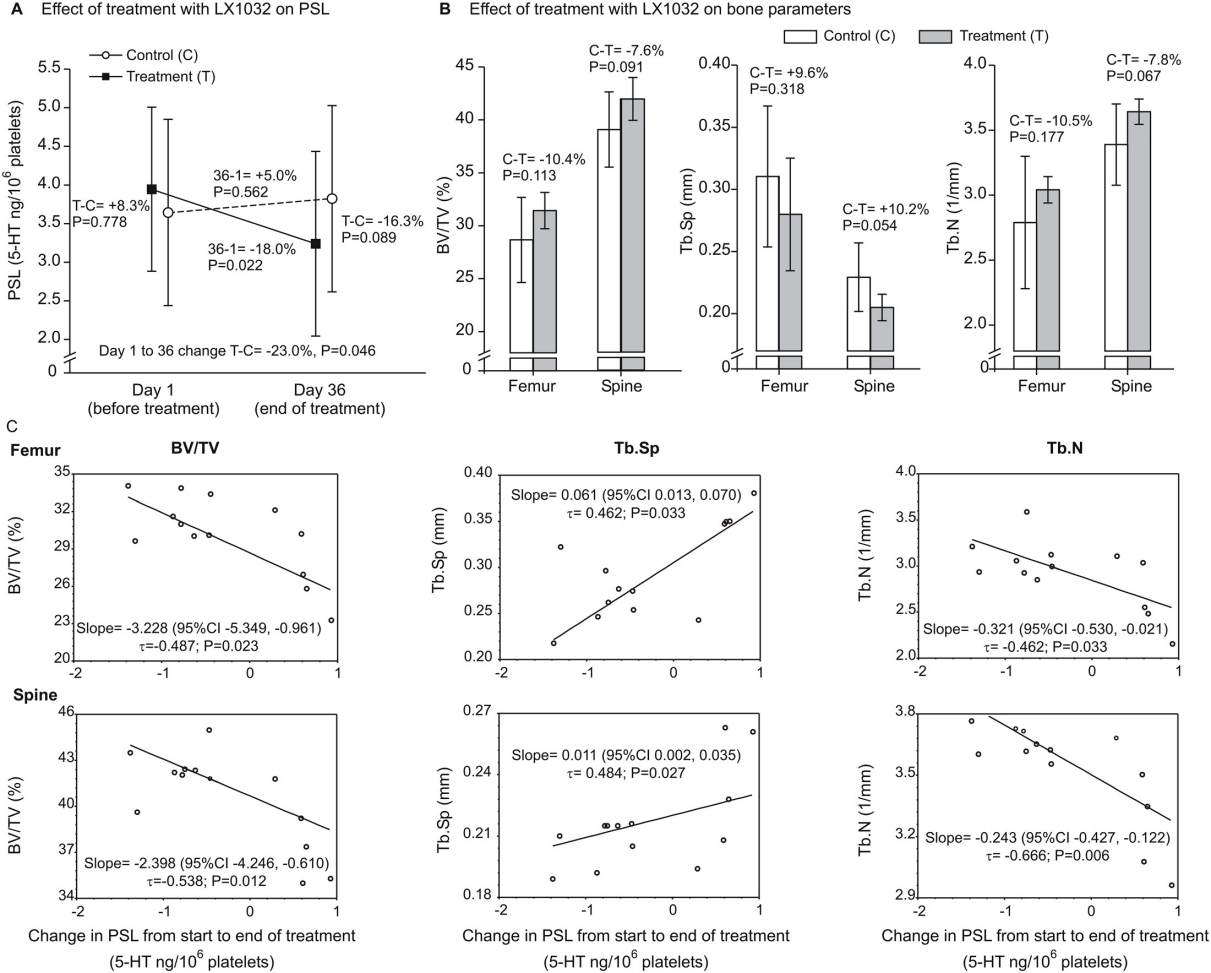
*In vivo* effects of *Tph1* inhibition on platelet serotonin levels (PSL) and bone parameters in 12 months old high 5-HT subline. On day 1 of the experiment, PSL was measured and treatment with LX1032 (25 mg/kg) (n = 7) or vehicle (control, n = 6) was commenced. At the last day of treatment (Day 36), PSL was determined again. Animals were sacrificed 24 h after the last dose and bone volume (BV/TV, %), trabecular spacing (TbSp, mm) and number of trabecules (Tb.N, 1/mm) were determined in the femur and spine using μCT. A. Data are geometric means (±geometric SD) of PSL values on Day 1 and Day 36. A general linear mixed model (treatment, day [random], treatment*day interaction) was fitted to ln(PSL) and differences (expressed as percentages derived from geometric means ratios) were determined: a) in PSL between the two groups on days 1 and 36; b) in PSL between days 36 and 1, within each group; c) in change in PSL from Day 1 to Day 36 (interaction term coefficient) between the two groups. Adjustment for multiple comparisons was by the simulation method. B. Data are means (±SD) by bone parameter by group, separately for the femur and spine. A separate general linear model was fitted to each of the six ln-transformed outcomes. Differences between groups are expressed as percentage differences (derived from geometric means ratios). C. Non-parametric (Kendall's) regression of bone parameters on change in PSL from the start to the end of treatment. Data depict median slope with confidence interval, Kendall's tau coefficient and P-value.

To further explore the impact of 5HT synthesis on bone metabolism, 12 months old high-5HT rats received LX1032, an TPH1-specific inhibitor, which was expected to reduce 5HT synthesis in the gut and consequently PSL. Treatment for 36 days reduced PSL in treated animals by 18%, while in untreated controls PSL was slightly increased by 5%, which resulted, at the end of the treatment, in a total PSL difference of 23% between treated and untreated animals at the end of the treatment (Fig 7A). The reduced PSL was parallelled by an increased bone volume and trabecular number, as well as by decreased trabecular separation; however, statistical significance was not reached when compared to untreated high-5HT animals (Fig 7B). However, when analysing each animal individually, a statistically significant relationship between a decreased PSL and increased bone parameters, including bone volume and trabecular number, both in femur and spine, were observed (Fig 7C). The experiment has been terminated after 36 days due to weight loss of treated rats, potentially caused by a daily continuous gastric application of LX1032.

### Primary cell cultures of osteoblasts and osteoclasts

**Fig 8:**
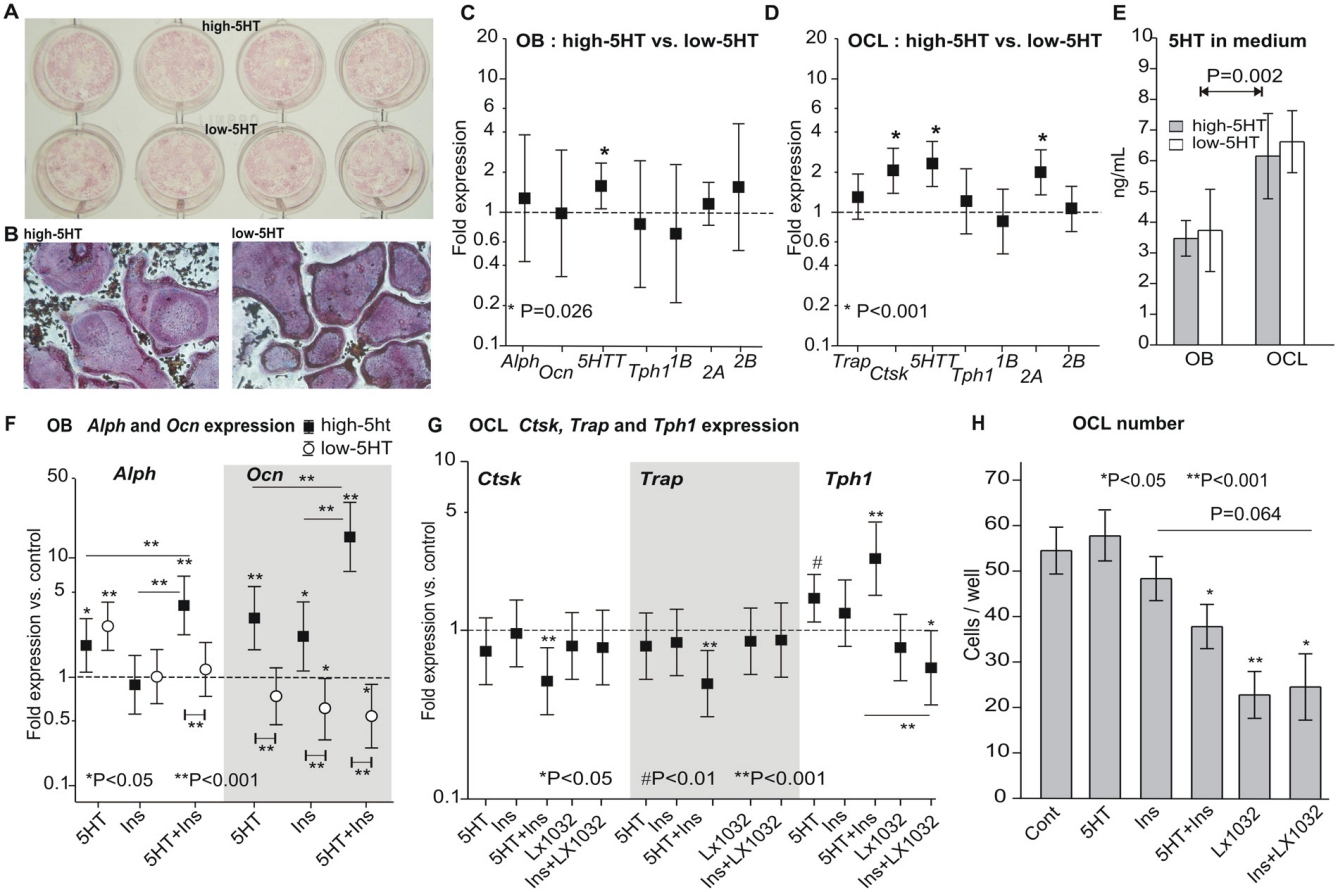
*In vitro* studies on primary rat osteoblasts and osteoclasts. A. Alkaline phosphatase staining of primary osteoblasts isolated from 5HT sublines. B. TRAP staining of primary osteoclasts isolated from 5HT sublines. C-D. Expression of mRNA for osteoblast (*Alph*, *Ocn*) and osteoclast (*Trap*, *Ctsk*) differentiation markers and 5HT-related molecules (*5HTT*, *Tph1*, *5HT*-*1B*, *-2A* and -*2B* receptors) in rat primary osteoblast (C) and osteoclast cultures (D) (n = 3–5). E. Levels of 5HT measured in media from primary high-5HT and low-5HT rat osteoblasts and osteoclasts (n = 4). F. Effects of added 5HT, insulin and their combination on *Alph* and *Ocn* expression from primary osteoblasts isolated from high-5HT and low-5HT rats (n = 3). G-H. Effects of added 5HT, insulin and LX1032 on *Ctsk*, *Trap*, and *Tph1* mRNA expression (G) (n = 3) and osteoclast number (H) (n = 4–7 wells analyzed per group) in primary osteoclast cultures from control Wistar rats. Expression data (C, D, F, G) are shown as fold difference over a control (indicated by the dashed line) with 95% confidence intervals. Stars (*) at estimates indicate significance vs. control, whereas horizontal lines and associated stars indicate significance in fold difference over control between treatments.

Cultured primary osteoblasts (Fig 8A) and osteoclasts (Fig 8B) from high-5HT and low-5HT rats expressed mRNAs for specific differentiation markers including alkaline phosphatase (*Alph*), osteocalcin (*Ocn*), tartrate resistant acid phosphatase (*Trap*) and cathepsin K (*Ctsk*), as well as 5HT-related molecules including *5HTT*, *Tph1* and *5HT* receptors *1B*, *2A* and *2B*. High-5HT rats showed a higher expression of *5HTT* in osteoblasts (Fig 8C) and higher *5HTT* and *5HT-2A* level in osteoclasts (Fig 8D). Expression of mRNA for *Ctsk* was higher in high-5HT osteoclasts (Fig 8D), while primary osteoblasts from two sublines did not differ in expression of *Alph* or *Ocn* (Fig 8C). Osteoclasts from both rat sublines released more 5HT into the medium than osteoblasts, without difference between the sublines (Fig 8E). Treatment of primary osteoblasts from both sublines with 5HT, insulin and their combination showed different effects on differentiation markers. Treatment with 5HT upregulated the expression of *Alph* in osteoblasts from both sublines, while *Ocn* expression was increased only in high-5HT osteoblasts. Insulin had no effect on *Alph* while *Ocn* was upregulated in high-5HT and downregulated in low-5HT osteoblasts. Simultaneous addition of 5HT and insulin had an additive effect on *Alph* and a synergistic effect on *Ocn* expression in high-5HT osteoblasts. In low-5HT osteoblasts there was no effect on *Alph* expression, while the combined treatment downregulated *Ocn* mRNA (Fig 8F). In primary bone marrow-derived osteoclasts, treatment with 5HT and the combination of 5HT and insulin significantly increased *Tph1* mRNA expression (Fig 8G). Interestingly, individual 5HT and insulin treatment of osteoclasts had no effect on the expression on *Trap* and *Ctsk* mRNA (Fig 8G), and on the osteoclast number (Fig 8H); however, the combined treatment with 5HT and insulin decreased both *Trap* and *Ctsk* mRNA expression (Fig 8G). Treatment with the specific TPH1 inhibitor, LX1032, significantly decreased the number of primary osteoclasts (Fig 8H). A similar effect was observed in the combined treatment with LX1032 and insulin (Fig 8G and 8H).

Our experiments further demonstrated that both osteoblasts and osteoclasts expressed the mRNA for key serotonergic elements (*5HTT*, *Tph1* and *5HT* receptors 1B, 2A and 2B) with significantly different expression in 5HTT between 5HT sublines. Cultured osteoblasts from high-5HT and low-5HT rat subline showed a different response to the combined treatment with 5HT and insulin, whereas inhibition of 5HT synthesis decreased the number of osteoclasts in culture.

## Discussion

To address the role of 5HT in bone we used selective breeding through several generations to develop a new animal model with constitutively high or low PSL caused by high or low level of PSU. Divergence of mean values of platelet 5HT parameters stabilized after 5 generations at about 70% (low-5HT) and 150% (high-5HT) of the mean value of the starting population. Our model is generated by selection for naturally occuring extreme PSL values and reflects the physiological conditions more closely than similar models generated by genetic manipulation. However, we are aware of its main limitation, i.e. lack of exact knowledge about genetical differences between the sublines which will be the subject of further analyses.

**Fig 9:**
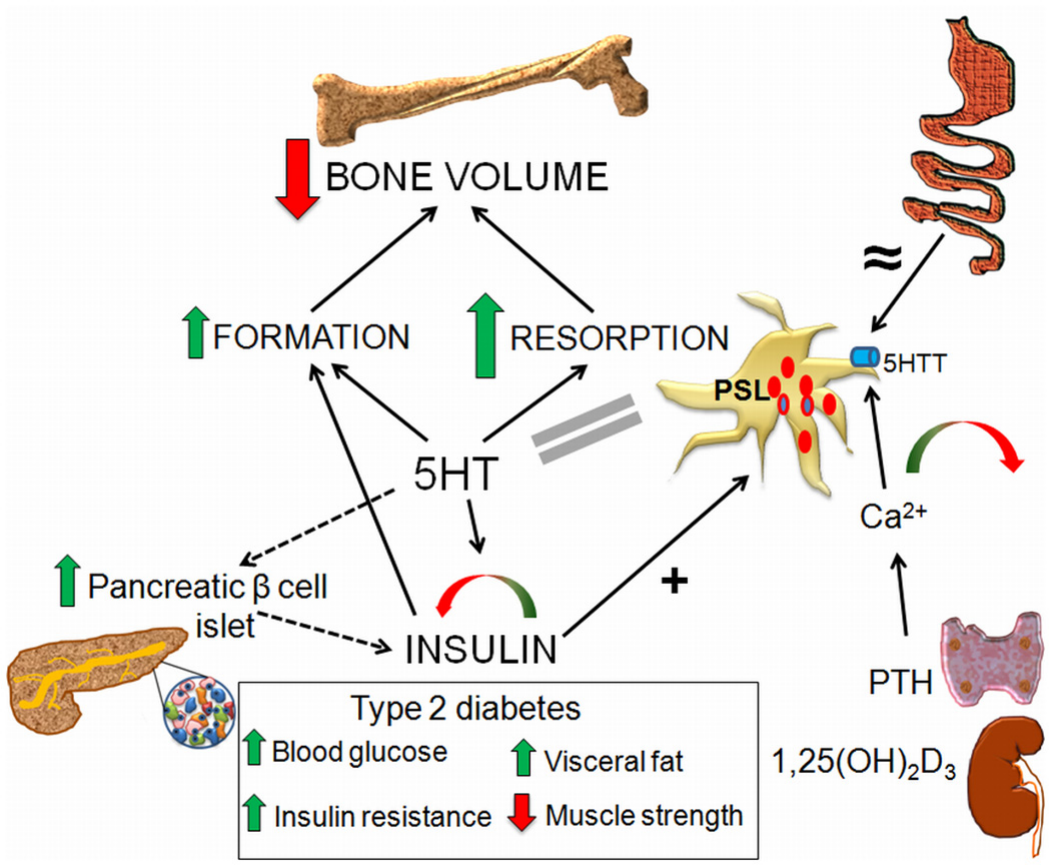
Systemic role of the peripheral 5HT. Gut synthesized 5HT enters the platelets via the 5HTT. The quantity of 5HT in platelets depends on the 5HTT activity, while the rate of 5HT synthesis in the gut is equal between both rat sublines (≈ sign). Changes in the serum Ca^2+^ level, influenced by PTH from parathyroid glands and by 1,25(OH)_2_D_3_ from the kidney, impact the platelet 5HTT activity, with a bidirectional effect on PSL (green-red arrow). Elevated 5HT bidirectionally influences the plasma insulin level (green-red arrow) and induces the hyperthrophy of pancreatic β-cells (dashed arrow), leading to type 2 diabetes with an increased plasma glucose, insulin resistance, glucose intolerance, visceral fat volume and decreased muscle strength. In return, plasma insulin level positively correlates with the PSL (+ sign). Increased insulin and 5HT have an additive effect on bone formation (green arrow). Elevated 5HT increases both bone formation and resorption (larger green arrow), thus increasing the bone turnover and resulting in the net bone loss (large red arrow). 5HT—serotonin, 5HTT—serotonin transporter, PSL—plasma serotonin level, PTH—parathyroid hormone.

In previous studies using genetically modified mice, investigators found that 5HT deficiency was either anabolic to bone with a high osteoblastic activity [1], that 5HT stimulated the osteoclastic bone resorption [2], or had no bone effect [3]. Analysis of bone remodeling in 5HT-deficient mice revealed that 5HT stimulated bone resorption in a cell autonomous manner, and suggested that 5HT acts locally in a bone serotoninergic microenvironment [7]. Mechanistically, it has been reported that the impact of *Lrp5* gain-of-function mutation had an impact on *Tph1* gut expression and lowered the blood 5HT level [1,2] and consequently increased the bone formation and bone mass accrual [10], however, this finding was not independently confirmed [3,11]. We demonstrated that constitutively high PSL in rats was associated with an increased bone turnover and a subsequent bone loss due to the increased bone formation and mineral apposition rate, paralleled by an increased number and function of osteoclasts compared to rats with significantly lower PSL. The bone loss was irreversible and more pronounced in aged rats. 5HT from platelets of high-5HT rats might influence the bone volume systemically by increasing the level of 5HT in the platelet free plasma, as previously suggested in mice [1], and/or indirectly via increased circulating level of insulin, calciotropic hormones (PTH, 1,25(OH)_2_D_3_), or growth factors (FGF23). However, complex biochemical profile of described circulating molecules could have influence on the local bone homeostasis, as we demonstrated that differentiation of both, osteoblasts and osteoclasts, is regulated by 5HT as well as by insulin (Figs 8 and 9).

To further explore the impact of 5HT on bone homeostasis, we performed an experiment where high-5HT animals were chronically treated with the specific TPH1 inhibitor, which lowered PSL after 36 days of treatment. The drop of PSL was parallelled by increased bone volume and trabecular number, while trabecular separation was decreased. This trend became significant when analyzed for each animal individually, which suggested that TPH1 inhibitor lowers PSL and the extent of change was directly associated by subsequent changes in the bone structure, confirming a direct relationship between TPH1 activity, PSL value and the bone volume. Based on our findings, we suppose that a prolonged treatment with TPH1 inhibitor (for example, several months) would have even more pronounced effect on the bone volume. This experiment has been stopped due to reduced solubility of the TPH1 inhibitor (LX1032) and increased volume of solvent used daily by gastric gavage which resulted in the weight loss of treated animals.

However, due to the experiment termination after 36 days and a limited number of rats included in the experiment with the TPH1 inhibitor, these results, although showing a significant correlation between PSL and bone parameters, should be considered as preliminary and more experimental work with high- and low-PSL animals, testing also other TPH1 inhibitors, should be conducted.

Low plasma 5HT was reported also in patients with high-bone-mass phenotype (HBM) owing to *Lrp5*-T2531 mutation [9], however, a subsequent study by other authors failed to find any correlation between 5HT and bone mass in HBM population [14]. At this point it is not clear whether 5HT synthesized by the osteoclastic cells or in the gut is playing a role in our model system. It should be noted that we did not find an increased 5HT production rate by either an over-expression of TPH1 or due to TPH1 hyperactivity. Rather, increased platelet 5HT level was due to increased uptake of free 5HT. These data suggest that in this study the increased bone remodeling observed in high-5HT rats was due to release of platelet 5HT locally during bone remodeling cycles. Alternatively, it is also possible that the up-regulated 5HTT in other cell systems, in addition to the platelets in our model, might lead to increased uptake and a subsequent increase in cytoplasmic levels of free 5HT in cell types such as the osteoclasts and osteoblasts. Cytoplasmic 5HT has been shown to play a critical role in regulating secondary signaling events and cell function via serotonylation of various proteins [29,30].

We are currently analyzing if platelet 5HT uptake in high-5HT rats is influenced solely by the number of functional transporters and whether loci responsible for the difference between the high- and low-5HT subline co-segregate with 5HTT gene, including traits that control bone mass and 5HT levels.

The extent of the elevated blood 5HT effect on the lumbar spine and the distal femur was found not to be equal. This trend has been repeatedly demonstrated in the patients with osteoporosis [31] or astronauts during long-duration spaceflight [32] where bone loss is more pronounced at the site of lumbar spine as compared to the hip or long bones. Detailed *in vivo* and *in vitro* experiments have also shown that these bone sites respond differently to specific treatment [33,34]. Our results show that elevated 5HT has a negative impact on the whole skeleton with site to site variations. Surprisingly, in the high-5HT rats, we also found significantly enlarged pancreatic β cells and hallmarks of a T2D phenotype. Increased adiposity, often associated with insulin resistance, was also seen in the high-5HT rats. While the exact etiology of the T2D phenotype in the high-5HT rats is unclear, 5HT has been reported to affect β cell mass and function. For example, in otherwise healthy animals, it has been previously demonstrated that 5HT during pregnancy regulates pancreatic β-cell mass due to energy requirements of the fetus and changes in maternal metabolism [35]. The enlargement of pancreatic β islets might reflect the peripheral insulin resistance as seen in T2D; however, 5HT has also been shown to modulate insulin secretion from pancreatic β cells via serotonylation of GTPases [29], further supporting the finding that elevated 5HT increases insulin release in high-5HT rats. Although the precise sequence of events that occurs in bone following the development of insulin resistance has not been resolved, evidence to date demonstrates that patients with T2D have lower serum bone formation markers, decreased muscle tone and reduced bone strength as evidenced by microindentation studies *in vivo* [36,37]. Finally, in contrast to what is observed in the high-5HT rats, TPH1 KO mice have markedly reduced circulating 5HT levels, insulin deficiency and an associated T1D phenotype [38].

Previously, it has been shown that 5HT or 5HTT deficiency in mice increased the body mass during growth [7,39]. On the contrary, we observed an increased body weight and bone length in high-5HT rats which might have been a consequence of enhanced growth hormone production in the pineal gland due to excess of 5HT [40]. We suggest that in rats calciotropic hormones, via changes of serum calcium, directly and indirectly effect the 5HT release from the gut enterochromaffin cells suggesting that systemically available 5HT from platelets, as well as from osteoblasts and osteoclasts effects the bone metabolism, and in combination with insulin synergistically upregulate bone cell activity. There was a higher expression and activity of *5Htt* mRNA in cultured osteoblasts and osteoclasts obtained from high-5HT rats. Since 5HT regulates osteoclast differentiation through 5HTT [41], different bone phenotype in 5HT sublines could be due to different 5HT level in the bone microenvironment.

As evidenced from our results, there is a relationship between serum calcium and PSL which was mediated by calciotropic hormones and insulin. Ageing *per se* independent of changes in vitamin D or renal function is associated with an increase in integrated PTH secretory response to changes in serum calcium [42]. Interestingly, the association between PSL and calcium arose from several experimental interventions in high-5HT rats, suggesting that calciotropic hormones affect bone, and can affect pancreas and eventually other organs. We, therefore, suggest that the 5HTT expression is regulated by calcium as a consequence of changing calciotropic hormone serum levels affecting plasma 5HT and activity of bone cells. In addition to these data it has been shown that activation of TPH1 requires calcium and calmodulin [30,43], which act through the phosphorylation of TPH1 on Ser58 and Ser260 [44]. In our animal model, blood concentration of 1,25(OH)_2_D_3_ was lower in high-5HT animals suggesting the role of vitamin D in 5HT production. There is also evidence that 1,25(OH)_2_D_3_ represses the transcription of TPH1 by suppressing the distal vitamin D-response element (VDRE) [45]. Thus, 5HT synthesis in the gut is likely to be under a complex influence of serum calcium, which stimulates the TPH1 activity, and serum 1,25(OH)_2_D_3_, that inhibits the *Tph1* gene transcription.

Interestingly, patients with a carcinoid syndrome (tumor releasing also 5HT) did not have altered bone volume [46,47]. However, it should be mentioned that in the carcinoid syndrome 5HT is not released in high concentration constantly, but in intermittent peaks. 5HIAA, a catabolic product of 5HT metabolism, which is usually determined in urine and is constantly increased in carcinoid syndrome, accumulates in the urine and its concentration is constantly high, but this does not apply for 5HT. Therefore, studies on carcinoid patients could not be directly compared with studies on animal model with constantly administered 5HT.

Collectively, increased PSL in rats was associated with a complex endocrine, paracrine and autocrine activities of various cells and organs leading to hypertrophy of β-pancreatic islets, increased plasma glucose and lipids and decreased 1,25(OH)_2_D_3_ that at least in part contributed to the increased remodeling rate and bone loss (Fig 9). This was supported by demonstration that treatment of high- and low-5HT rats with PTH, 1,25(OH)_2_D_3_, SSRI and streptozotocin, influenced PSL, but also plasma insulin, both directly and via changes of serum calcium concentration. The results of these experiments suggested that bone phenotype deduced from 5HT deficient mice cannot be directly translated into high-5HT PSL phenotype in rats. Our data in accordance with the published data indicate that 5-HT levels directly impacts osteoclastic bone resorption [7]. Some of the differences between our data and the published literature could simply arise from differences in 5HT metabolism (low levels due to deletion of TPH1 vs. increase PSL levels due to 5HTT upregulation) and/or due to differences in bone metabolism between the mouse and rat [48]. These findings suggested an association between the increased PSL, T2D phenotype and bone loss in a complex systemic environment composed of unbalanced calciotropic hormones and calcium influence on the 5HT metabolism (Fig 9).

## Supporting Information

S1 Dataset Model description dataset. (XLSX)

S2 Dataset CT and histomorphometry dataset. (XLSX)

S3 Dataset Biochemistry dataset. (XLSX)

S4 Dataset Diabetes dataset. (XLSX)

S5 Dataset Pharmacology dataset. (XLSX)

S6 Dataset TPH1 inhibition dataset. (XLSX)

S7 Dataset In vitro dataset. (XLSX)
